# Characterizing a Halo-Tolerant GH10 Xylanase from *Roseithermus sacchariphilus* Strain RA and Its CBM-Truncated Variant

**DOI:** 10.3390/ijms20092284

**Published:** 2019-05-09

**Authors:** Seng Chong Teo, Kok Jun Liew, Mohd Shahir Shamsir, Chun Shiong Chong, Neil C. Bruce, Kok-Gan Chan, Kian Mau Goh

**Affiliations:** 1 Faculty of Science, Universiti Teknologi Malaysia, Skudai 81310, Johor, Malaysia; scteo4@gmail.com (S.C.T.); kokjunliew@gmail.com (K.J.L.); shahir@utm.my (M.S.S.); cschong@utm.my (C.S.C.); 2 Centre for Novel Agricultural Products, Department of Biology, University of York, Wentworth Way, York YO10 5DD, UK; neil.bruce@york.ac.uk; 3 Division of Genetics and Molecular Biology, Institute of Biological Science, Faculty of Science, University of Malaya, Kuala Lumpur 50603, Malaysia; 4 International Genome Centre, Jiangsu University, Zhenjiang 212013, China

**Keywords:** glycoside hydrolase, xylanase, carbohydrate-binding module, CBM truncation, halo-tolerant, xylan hydrolysis

## Abstract

A halo-thermophilic bacterium, *Roseithermus sacchariphilus* strain RA (previously known as *Rhodothermaceae* bacterium RA), was isolated from a hot spring in Langkawi, Malaysia. A complete genome analysis showed that the bacterium harbors 57 glycoside hydrolases (GHs), including a multi-domain xylanase (XynRA2). The full-length XynRA2 of 813 amino acids comprises a family 4_9 carbohydrate-binding module (CBM4_9), a family 10 glycoside hydrolase catalytic domain (GH10), and a C-terminal domain (CTD) for type IX secretion system (T9SS). This study aims to describe the biochemical properties of XynRA2 and the effects of CBM truncation on this xylanase. XynRA2 and its CBM-truncated variant (XynRA2ΔCBM) was expressed, purified, and characterized. The purified XynRA2 and XynRA2ΔCBM had an identical optimum temperature at 70 °C, but different optimum pHs of 8.5 and 6.0 respectively. Furthermore, XynRA2 retained 94% and 71% of activity at 4.0 M and 5.0 M NaCl respectively, whereas XynRA2ΔCBM showed a lower activity (79% and 54%). XynRA2 exhibited a turnover rate (*k*_cat_) of 24.8 s^−1^, but this was reduced by 40% for XynRA2ΔCBM. Both the xylanases hydrolyzed beechwood xylan predominantly into xylobiose, and oat-spelt xylan into a mixture of xylo-oligosaccharides (XOs). Collectively, this work suggested CBM4_9 of XynRA2 has a role in enzyme performance.

## 1. Introduction

Xylan is one of the most abundant polymers in plant biomass. The polymer consists of a β-1,3/1,4-linked xylopyranose backbone with side branches such as *O*-acetyl, α-4-*O*-glucuronic acid, α-l-arabinofuranose, *p*-coumaric acid, or ferulic acid at C-2 or C-3 positions [1]. Due to such complexity, a complete hydrolysis of xylan requires synergism of various xylanolytic enzymes including endo-β-1,4-d-xylanase, β-d-xylosidase, α-d-glucuronidase, α-l-arabinofuranosidase, and acetylesterase [2]. Among these enzymes, endo-β-1,4-xylanase (E.C. 3.2.1.8) plays a crucial role in hydrolyzing the β-1,4-glycosidic bonds of the xylan to form xylo-oligosaccharides (XOs) and xylose. According to the Carbohydrate-Active Enzyme (CAZy) database (http://www.cazy.org) [3], endo-β-1,4-xylanases are currently grouped in glycosyl hydrolase (GH) 5, 8, 10, 11, 30, 43, 51, 98, and 141 families. The majority of endo-β-1,4-xylanases belong to GH10 and GH11 families, which are distinctive of their respective origin, molecular properties, and protein structure [4].

Xylanases are produced by a diverse range of organisms, which include fungi, bacteria, yeast, algae, protozoa, crustaceans, and insects. Fungal and bacterial xylanases are important due to their superior properties, which could potentially be applied in industrial processes [5]. As summarized in a review article [6], xylanases are utilized for the delignification of paper pulp, modification of cereal food, improvement of digestibility of animal feedstock and production of xylo-oligosaccharides for pharmaceutical industries. Selection of the types of xylanases for these applications is based on suitability. For instance, thermostable alkaline xylanases are applicable for efficient biobleaching of pulp and paper [7], while thermostable acidic xylanases are applicable for animal feed processes [8]. Xylanases which are active and stable in low or high pH values are suitable for hemicellulosic biomass saccharification [5]. Xylanases with an optimum activity at low temperature and alkaline pH are applicable in detergent formulation additives [9]. Earlier reports suggested that xylanases obtained from psychrophilic species could improve the quality of bread and fruit juices [10,11]. Xylanases from halophilic microorganisms often exhibit salt-tolerance, which can be used for wastewater treatment and marine/saline food preparation [12].

The protein architecture of endo-β-xylanases comprises a glycoside hydrolase catalytic domain that is sometimes associated with one or more carbohydrate-binding modules (CBMs) [13]. Endo-β-xylanases without a CBM have also been reported [14,15,16,17]. CBMs do not contribute directly to catalytic mechanisms. However, they play a role in carbohydrate recognition and binding. The presence of CBM binding allows carbohydrate-active enzymes to concentrate on the polysaccharide surface and improve the overall catalytic efficiency [18]. There are currently 84 families of CBMs. These CBMs display considerable variations in substrate specificity against crystalline cellulose, non-crystalline cellulose, chitin, β-1,3/1,4-glucans, starch, glycogen, xylan, mannan, galactan, and inulin [19]. In the CAZy database, various families of CBMs were appended with GH10 xylanases, predominantly from GH 1, 2, 3, 4, 6, 9, 13, and 22, as well as from GH 10, 15, 35, and 37.

A rare halo-thermophilic bacterium, initially designated as *Rhodothermaceae* bacterium RA (NCBI taxonomy ID: 1779382), was isolated from a hot spring in Langkawi (6°25′22.31″ N, 99°48′48.97″ E), Malaysia [20]. The bacterium exhibited a low identity of 16S rRNA (89.3%) and ANI value (79.3) to the closest strain *Rhodothermus marinus* DSM 4252^T^. This information indicates that *Rhodothermaceae* bacterium RA might represent a new genus in the family *Rhodothermaceae* [20,21]. In 2019, Park et al. reported a strain MEBiC09517^T^ isolated from a port in South Korea [22]. MEBiC09517^T^ was proposed as the first member of the new genus and the authors suggested that the strain be classified as *Roseithermus sacchariphilus* gen. nov., sp. nov. Due to high similarity (ANI value of 96.2%) between *Rhodothermaceae* bacterium RA and strain MEBiC09517^T^, we propose that our strain is a subspecies of *Roseithermus sacchariphilus*. To differentiate both strains, we renamed our bacterium *Roseithermus sacchariphilus* RA. In this study, a xylanase gene (*xynRA2*) was cloned from this bacterium. The study aims to describe the biochemical properties of this enzyme and to understand the effects of CBM truncation on the xylanase.

## 2. Results and Discussion

### 2.1. Bioinformatic Analysis

Numerous xylanases have been discovered from extreme habitats such as soda lakes, marine sediments, and hot springs [23,24,25]. We previously isolated *R. sacchariphilus* RA from a saline hot spring (45 °C, pH 7.1, 13,000 mg/L for Cl^−^ ion, and 7900 mg/L for Na^+^) [26]. A complete genome sequencing elucidated that strain RA harbors 57 GHs affiliated to 30 families [21]. Two non-homologous xylanases were identified and designated as XynRA1 and XynRA2 respectively. The xylanase XynRA1 (Genbank: ARA95075.1) has 379 amino acids and lacks a CBM, while the xylanase XynRA2 (Genbank: ARA92359.1) consists of 813 amino acids and a CBM. XynRA2 was chosen for further study, as we were interested in the function of the CBM attached to this xylanase.

**Figure 1:**
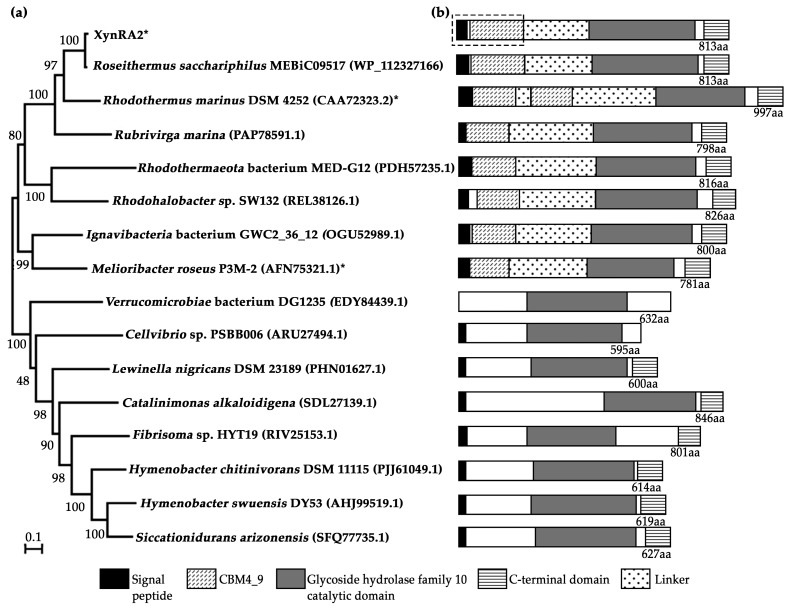
(**a**) Protein dendrogram of XynRA2 and its close homologs. The signal peptide sequences of the proteins were not included in the phylogenetic analysis. Asterisks (*) denote xylanases which have been characterized, otherwise represent genome annotated xylanases. (**b**) Schematic domains arrangement of the respective proteins identified by InterPro. Dotted-line box represents the truncated region in XynRA2ΔCBM.

XynRA2 and the putative xylanase annotated in the genome of *R*. *sacchariphilus* strain MEBiC09517^T^ are homologs with the identity of 98.6%. The protein sequence of XynRA2 has the identity of 50–65% with xylanases from *Rubrivirga marina*, *Verrucomicrobiae* bacterium DG1235, *Lewinella nigricans*, *Catalinimonas alkaloidigena*, *Fibrisoma* sp. HYT19, *Rhodohalobacter* sp. SW132, *Cellvibrio* sp. PSBB006, and *Ignavibacteria* bacterium GWC2_36_12. The xylanase XynRA2 shares less than 50% identity with xylanases from *Rhodothermaeota* bacterium MED-G12, *Hymenobacter chitinivorans*, and *Siccationidurans arizonensis*. The xylanases mentioned earlier including that of *R*. *sacchariphilus* MEBiC09517^T^ were deduced from genome annotation and have not been heterologously expressed and characterized. A phylogenetic tree utilizing a neighbor-joining algorithm was built to show a relationship of XynRA2 with selected counterparts (Figure 1a). In comparison to the well-characterized xylanases, XynRA2 is 73.1% in identity to that of xylanase Xyn10A produced by *R*. *marinus* DSM 4252 and 52.3% to a xylanase Xyl2091 from *Melioribacter roseus* P3M-2 [27,28]. The sequence identity is low between XynRA2 to other well-studied enzymes, including xylanases that are from *Fusarium graminearum* (38%) [29], *Trichoderma reesei* QM6α (28%) [30], *Bacillus stearothermophilus* T-6 (26%) [31], and *Thermoanaerobacterium saccharolyticum* B6A-RI (25%) [32].

**Figure 2:**
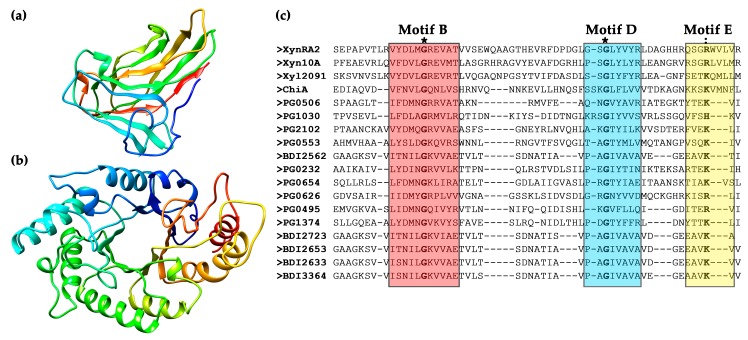
Putative structure of (**a**) CBM4_9 and (**b**) GH10 catalytic domain of XynRA2. The models were colored with the rainbow scheme (blue N-terminus, follows by green, yellow, and red C-terminus); (**c**) multiple sequence alignment of XynRA2 CTD with the counterpart of Xyn10A from *R*. *marinus*, Xyl2019 from *M*. *roseus*, ChiA from *F*. *johnsoniae*, as well as CTD proteins from *P*. *gingivalis* and *Parabacteroides distasonis*. Amino acid stretch for Motif B, D, and E are indicated by red, blue, and yellow boxes, respectively. Asterisks (*) indicate fully conserved amino acids while colon (:) indicates amino acid groups of similar properties.

A mature XynRA2 protein sequence comprises a CBM (Gln^36^–Asn^198^), a catalytic domain (Glu^396^–Tyr^712^), and a CTD (Trp^735^–Val^810^) (Figure 1b). The homology model of CBM clearly denoted the β-sandwich structure formed by eleven anti-parallel β-strands, while that of the catalytic domain is a typical TIM-barrel consisting of eight alternating β-strands and β-helices (Figure 2a,b). Similar to xylanases from *R*. *marinus* (Xyn10A) and *M*. *roseus* (Xyl2091), these enzymes possess a GH10 catalytic domain. From the multiple sequence alignment with six GH10 xylanases with crystal structures, the putative catalytic residues for XynRA2 were identified as Glu^520^ and Glu^635^. The linker region connecting the GH10 domain and CBM comprises 198 residues.

It is likely that in the wild-type *R. sacchariphilus* RA, XynRA2 is exported across the cytoplasmic membrane by the Sec pathway due to the presence of a signal peptide (Met^1^ to Ala^33^). In addition, XynRA2 has a CTD that enables the protein to be secreted across the outer membrane by T9SS. The T9SS protein secretion pathway is also known as Por secretion system (PorSS) [33,34] which was discovered in *Porphyromonas gingivalis* for secreting a potent protease gingipains [34]. Besides being identified in *R. sacchariphilus* RA, we noticed that in another genome sequencing project, some other annotated cellulases and hemicellulases incorporated a CTD; however, there has been little research describing the actual function of T9SS to GH enzymes. The two closest homologs of XynRA2, the Xyn10A from *R. marinus* and Xyl2091 from *M. roseus* also possessed a similar CTD [28,35]. The CTD possesses five short motifs, in which Motif B, Motif D and Motif E are important for the extensive modification by T9SS [36,37]. By aligning the CTD region of XynRA2 with other xylanases, the well-conserved Gly residues were identified in Motif B and Motif D, whereas Arg substituted the almost-conserved Lys in Motif E [37] (Figure 2c). Proteins that possessed the CTD were found to be cell-anchoring or rely on CTD for secretion, such as Xyn10A from *R*. *marinus* DSM 4252 as well as SprB, RemA, and ChiA from *Flavobacterium johnsoniae* [35,36]. Collectively, this suggests that XynRA2 could be a cell-anchoring enzyme. However, further experimental validation is required.

Based on an InterPro analysis, the CBM of XynRA2 was annotated as CBM4_9. The closest biochemically characterized xylanase (Xyn10A) from *R*. *marinus* DSM 4252 has two dissimilar CBM4_9s arranged in tandem (Figure 1b), which were denoted as “CBM4-1” and “CBM4-2” in the original article [38]. Another close homolog, a characterized xylanase (Xyl2091) from *M*. *roseus*, also possessed a CBM4_9 [28]. Interestingly, the amino acid stretch of the CBM4_9 from *R. sacchariphilus* RA is only 70% and 51% identical to *R*. *marinus* and *M*. *roseus* counterparts respectively, suggesting that the affinity of the three enzymes against hemicellulose might be different. Different families of CBMs such as CBM6_36 for XynG1-1 [39], CBM13 for XynAS27 [40], and dual CBM9-CBM22 for XynSL3 [24] were often reported in GH10 xylanases. According to the CAZy, other CBMs associated with xylanases are from families 1, 2, 3, 10, 15, 35, and 37. The CBM4 family from xylanases usually binds to xylan β-glucan, and/or amorphous cellulose [41,42]. We anticipated the substrate specificity of CBM4_9 in XynRA2 to be similar. Several reports have shown that the removal of the CBMs affected the biochemical properties of their partnering xylanases [39,40,43]. Therefore, we constructed a mutant enzyme (designated as XynRA2ΔCBM) by deleting the CBM4_9 but retaining the linker connecting the CBM to the catalytic domain to evaluate the effect of its truncation on the xylanase.

### 2.2. Expression of Recombinant XynRA2 and XynRA2ΔCBM

**Figure 3:**
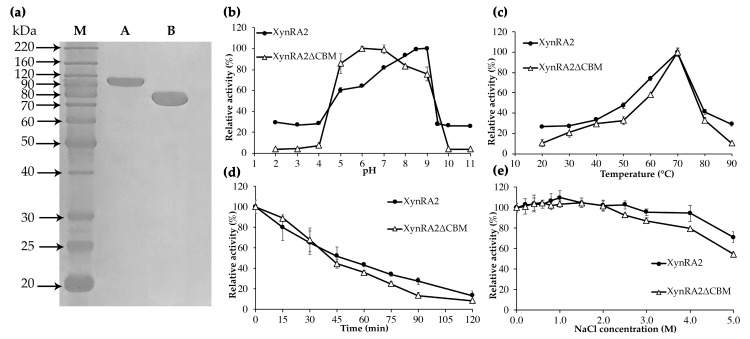
Molecular properties of purified XynRA2 and XynRA2ΔCBM. (**a**) SDS-PAGE analysis of purified XynRA2 and XynRA2ΔCBM. **M**, BenchMark^™^ Protein Ladder; **A**, purified XynRA2; **B**, purified XynRA2ΔCBM; (**b**) effect of pH in the range of 2–11; (**c**) effect of temperature from 20–90 °C; (**d**) thermostability at 70 °C across 120 min; (**e**) effect of NaCl from 0–5.0 M concentration.

The gene fragments encoding for mature XynRA2 (2349 bp) and XynRA2ΔCBM (1857 bp) were cloned in pET28a(+), expressed in *E. coli* BL21 (DE3) and purified using Ni-NTA columns. The purified enzymes migrated as two distinct bands around 90 kDa and 70 kDa on SDS-PAGE, which were consistent with the theoretical molecular weight of XynRA2 (89.5 kDa) and XynRA2ΔCBM (68.5 kDa) respectively (Figure 3a).

### 2.3. Biochemical Characterization of XynRA2 and XynRA2ΔCBM

#### 2.3.1. Effect of pH and Temperature

Using beechwood xylan as the substrate, the purified XynRA2 had maximum activity at pH 8.5 and retained a relatively high activity between pH 7–9. Truncation of the CBM broadened the pH profile (pH 5–9) with the optimum pH shifted to 6.0 (Figure 3b). Similarly, CBM4_9 truncation changed the optimum pH from 7.5 to 7.0 for a xylanase PX3 from *Paenibacillus terrae* HPL-003 [44]. The working pH for the mutant PX3 also narrowed to pH 5–10, while the native PX3 had an active pH ranging from 3–12. The optimum pHs of xylanase Xyn10A from *R*. *marinus* and Xyl2091 from *M*. *roseus* were 7.5 and 6.5 respectively [28,45], while that of truncated counterparts was not reported.

The optimum temperature for the activity of native XynRA2 and XynRA2ΔCBM was 70 °C. Overall, the temperature profiles for both enzymes were identical (Figure 3c). To evaluate the thermostability, XynRA2 and XynRA2ΔCBM were incubated at 70 °C without substrate for a specific interval prior to measuring residual activity. The half-life of both XynRA2 and XynRA2ΔCBM at 70 °C was approximately 45 min; however, XynRA2ΔCBM was more sensitive to prolonged temperature treatment (Figure 3d). The optimum temperatures of Xyn10A and Xyl2091 were 80 °C and 65 °C respectively and their half-lives were about 90 min (80 °C) and 160 min (60 °C), respectively. Truncation of the CBM in Xyn10A from *R*. *marinus* also resulted in a decrease in thermostability, indicating that the CBM with this xylanase also contributed to enzyme stability [46]. Truncation of the CBM from xylanases from *Streptomyce rochei* L10904 (Srxyn10) [43], *Paenibacillus campinasensis* G1-1 (XynG1-1) [39], and *Streptomyces* sp. S27 (XynAS27) [40] showed that removal of the CBM did not affect the optimum temperature of xylanases. However, the truncated versions of XynG1-1 and XynAS27 displayed a significant decrease in thermostability [39,40]. In contrast, the CBM-truncated variant of Srxyn10 from *S*. *rochei* L10904 exhibited a substantial increase in thermostability at 60–70 °C despite sharing similar optimum temperature with its native counterpart [43]. 

#### 2.3.2. NaCl Tolerance

The *R. sacchariphilus* RA was capable of growing in media containing a high concentration of NaCl [20]. Since XynRA2 is probably expressed as an extracellular cell-bound enzyme, we decided to investigate the effect of NaCl on xylanase activity. Multiple xylanases are known to exhibited moderate halo-tolerance, but only limited reports have demonstrated extreme halo-tolerance ability as displayed by XynRA2 (Table 1). The relative activity of XynRA2 and XynRA2ΔCBM was slightly enhanced when the catalytic reactions were supplemented with 1.0 M NaCl. Notably, XynRA2 retained 94% of initial activity at 4.0 M, and 71% at 5.0 M NaCl. Although the mutant XynRA2ΔCBM was more salt-sensitive, the enzyme retained the relative activity of 79% at 4.0 M and 54% at 5.0 M (Figure 3e). The reason for the lower halo-tolerance is unknown. In addition, there is a lack of literature elucidating the relationship between CBM and halo-xylanase activity.

**Figure 4:**
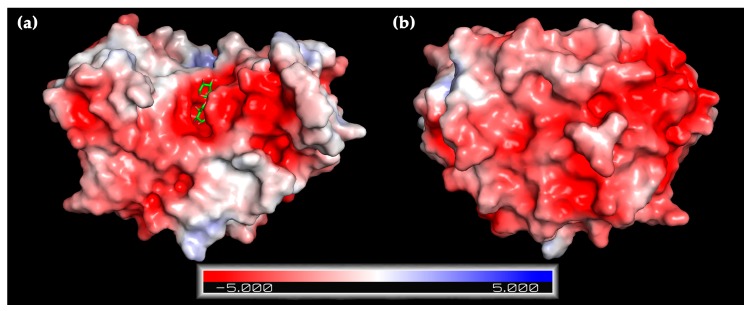
Predicted electrostatic potentials on the surface of XynRA2 catalytic domain from (**a**) top and (**b**) bottom views. The ligand (xylobiose) is bound at the catalytic binding pocket of XynRA2 as indicated in (**a**). Red and blue indicate negative and positive electrostatic potentials respectively.

A homology model of XynRA2 catalytic domain demonstrated a high distribution of acidic amino acids on the protein surface resulting in an overall negative electrostatic potential (Figure 4), which might explain the excellent protein stability in high NaCl concentration. Theoretically, halo-tolerant enzymes contain more acidic residues (Asp and Glu) than non-polar residues (Val, Ile, Leu, Met, and Phe). Halo-tolerant enzymes are also enriched with small residues (Ala, Val, Ser, and Thr) but lack Lys residue [47]. It has been proposed that excess acidic residues could facilitate the weakening of hydrophobicity or strengthening of hydrophilic forces on the enzyme surface, which increases water-binding capacity and prevent proteins aggregation at high salt concentration [48,49]. 

#### 2.3.3. Enzyme Kinetics

The specific activities and the turnover rate (*k*_cat_) of the purified XynRA2 and XynRA2ΔCBM were determined by reacting the enzymes with soluble beechwood xylan. The specific activities of XynRA2 and XynRA2ΔCBM were 300 U/mg and 160 U/mg respectively. The *k*_cat_ of the native and mutant enzymes were 24.8 s^−1^ and 15.7 s^−1^, respectively. We found that the truncation of CBM significantly affected the performance of the enzymes. This finding was in consistent with XynG1-1 from *P*. *campinasensis* that CBM truncation reduced the *k*_cat_ by 20% [39]. Removal of CBM alone did not affect the *k*_cat_ of XynAS27 from *Streptomyces* sp. S27. However, truncating CBM together with the linker reduced *k*_cat_ value by 25% [40]. On the other hand, the xylanase variant of Srxyn10 with a CBM truncation had a three-fold higher specific activity on beechwood xylan than its native counterpart [43].

#### 2.3.4. Substrate and Product Specificities

The purified XynRA2 and XynRA2ΔCBM were incubated with various substrates before analyzing them using HPLC. Generally, XynRA2 and XynRA2ΔCBM showed similar substrate specificities. Both enzymes were active on beechwood xylan, oat-spelt xylan, and xylo-oligosaccharides (XOs) such as X_6_, X_5_, X_4_ but not on X_3_ and X_2_. Except for xylose-based carbohydrates, the enzymes were unable to hydrolyze glucose-, maltose-, and arabinose-derived polymers such as carboxymethylcellulose (CMC), Avicel™, starch, pullulan, d-cellobiose, and arabinan. The results indicated that the enzymes did not possess either a cellulase or arabinase activity, suggesting that XynRA2 is a specific GH10 xylanase. This is in agreement with a recent statistical study that showed most of the characterized GH10 xylanases were mono-specific (96.8%, *n* = 350) towards xylanosic substrates [4].

**Figure 5:**
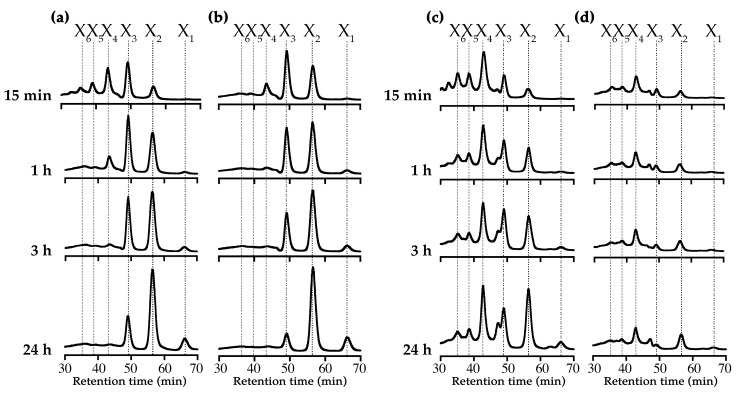
Product analysis of xylan degradation. (**a**,**b**) products formed from hydrolysis of beechwood xylan by XynRA2 and XynRA2ΔCBM, respectively; (**c**,**d**) products formed from hydrolysis of oat-spelt xylan by XynRA2 and XynRA2ΔCBM, respectively. The product peaks shown in this figure were normalized.

We compared the product formation pattern of XynRA2 and XynRA2ΔCBM by reacting the purified enzymes with beechwood xylan and oat-spelt xylan (Figure 5). Upon reacting XynRA2 with beechwood xylan, the products constituted a mixture of XOs ranging X_6_, X_5_, X_4_, X_3_, and X_2_ at the beginning of the reaction (15 min). After a prolonged hydrolysis (24 h), xylobiose (X_2_) was accumulated as the primary product together with detectable X_3_ and X_1_ (Figure 5a), and the product formation pattern for XynRA2ΔCBM against beechwood xylan was shown in Figure 5b. For reactions of 15 min and 24 h, the product profile for XynRA2ΔCBM was similar to that of XynRA2. Yet, the ratio of X_3_ and X_2_ was slightly different in the 1 h and 3 h reactions. Previous reports on xylanase rXTMA from *Thermotoga maritima* and xylanase A from *Caldibacillus cellulovorans* also showed a variation in the profiles of XOs produced by native and CBM-depletion xylanases [55,56].

Although the same reaction conditions were used with oat-spelt xylan, we obtained lower sugar yields, probably due to the physical structure of oat-spelt xylan which consisted of both insoluble and soluble fractions. Furthermore, the product profiles were also different for beechwood xylan and oat-spelt xylan. After prolonged reaction, X_4_, X_3_, and X_2_ were accumulated as the major products (Figure 5c). Interestingly, oat-spelt xylan was a poor substrate for XynRA2ΔCBM (Figure 5d), as reported for other xylanases [39,40,57,58]. A lower activity against oat-spelt xylan might be due to the inefficient binding onto the substrate, as a result of CBM truncation in XynRA2ΔCBM. It has also been recurrently reported that the truncation of CBMs affects catalytic efficiency of GHs towards other insoluble substrates but not the soluble counterparts [18,19].

## 3. Materials and Methods

### 3.1. Sequence Analysis

The gene sequence of putative xylanase (GenBank: ARA92359.1) was extracted from the complete genome of *R. sacchariphilus* RA (GenBank: CP020382.1) [20,21]. The mature xylanase gene was designated as *xynRA2*. The protein sequence similarity was assessed using NCBI BLASTp program (https://blast.ncbi.nlm.nih.gov/Blast.cgi). Multiple sequence alignments were performed using Clustal Omega (https://www.ebi.ac.uk/Tools/msa/clustalo/). A phylogenetic tree of XynRA2 with its closest homologs was constructed with the neighbor-joining algorithm using MEGA v7.0 software [59] with a bootstrap value of 1000. The signal peptide sequence was predicted using SignalP v4.1 (http://www.cbs.dtu.dk/services/SignalP/). Conservative domains were identified using InterPro. The homology model of XynRA2 was performed using SWISS-MODEL (https://swissmodel.expasy.org/) with an evolved CBM of Xyn10A from *R*. *marinus* (PDB: 3JXS) [60] and GH10 xylanase (XynB) from *Xanthomonas axonopodis* pv *citri* (PDB: 4PN2) complexed with xylotriose as a template [61]. The predicted model and its surface electrostatic potential were assessed using APBS plugin in PyMOL (v2.2.3, Schrödinger Inc., New York, NY, USA).

### 3.2. Cloning of Xylanases

Genomic DNA of *R. sacchariphilus* RA was extracted using DNeasy Blood & Tissue Kit (Qiagen, Hilden, Germany). The gene sequence of *xynRA2* was amplified from the genomic DNA using a forward primer GH10F (5′-AGCCATATGCGTGCGCAGAGCAACACCA-3′) and a reverse primer GH10R (5′-CGATGGGTACTGGTCCGCCTCGAGCACC-3′). The underlined sequences represent *Nde*I and *Xho*I restriction sites respectively. N-terminal signal peptide was not included in the recombinant enzymes. The truncated gene *xynRA2ΔCBM* was amplified using primer GH10F-LC (5′-AGCCATATGCCCCTGGCGGGAGC-3′) and GH10R.

Both the gene fragments were amplified using Q5^®^ High-Fidelity PCR kit (NEB, Ipswich, MA, USA). The PCR products were digested with *Nde*I and *Xho*I followed by ligation into pET28a(+) (Novagen, Madison, WI, USA) at the corresponding sites. The recombinant plasmids (pET28a_*xynRA2 and* pET28a_*xynRA2ΔCBM*) were separately transformed into *E. coli* BL21 (DE3) competent cells using the heat shock method. The transformants were grown in Luria-Bertani (LB) medium containing 50 μg/mL kanamycin at 37 °C for 18 h. Transformants harboring the recombinant plasmid were identified by restriction digestion and DNA sequencing. 

### 3.3. Expression and Purification of Xylanases

The transformed cells were grown in LB medium containing 50 μg/mL kanamycin at 37 °C to an *A*_600 nm_ of 0.6. Protein expression was induced by addition of isopropyl-β-d-thiogalactopyranoside (IPTG) at a final concentration of 0.4 mM at 25 °C for 18 h. The cells were harvested by centrifugation (6000× *g*, 4 °C, 10 min) and lysed using B-PER™ Direct Bacterial Protein Extraction Kit (Thermo Scientific, Waltham, MA, USA) The crude enzyme was collected (12,000× *g*, 4 °C, 10 min) and dialyzed against 20 mM sodium phosphate buffer (pH 7.4) at 4 °C overnight in a SnakeSkin™ Dialysis Tubing 10k MWCO (Thermo Scientific, Waltham, MA, USA). To purify the His-tagged proteins, the crude enzyme was loaded onto a Ni-NTA Superflow column (Qiagen, Hilden, Germany) equilibrated with 20 mM sodium phosphate buffer (pH 7.4) and 50 mM imidazole. The enzymes were eluted with a linear gradient of 50–500 mM imidazole in 20 mM phosphate buffer (pH 7.4) containing 500 mM NaCl. Upon elution, fractions containing the XynRA2 and XynRA2ΔCBM were respectively pooled and dialyzed against 20 mM sodium phosphate buffer (pH 7.4) at 4 °C overnight to remove the remaining salts. The purity and apparent molecular mass of XynRA2 and XynRA2ΔCBM were validated by SDS-PAGE. The activity of the purified enzymes was assayed as described below.

### 3.4. Xylanase Assay

The xylanase activity of XynRA2 and XynRA2ΔCBM was calculated by measuring the reducing sugars released from substrates using 3,5-dinitrosalicylic acid (DNS) method. The reaction mixtures contained 50 μL of appropriately diluted enzymes and 500 μL of 1% (*w*/*v*) beechwood xylan (Megazyme, Bray, County Wicklow, Ireland) in 0.1 M Tris-HCl buffer (pH 8.5). The enzymatic reaction was carried out at 70 °C for 15 min, stopped with 500 μL DNS reagent and boiled for 5 min. The absorbance at 540 nm was measured when the reaction mixture is cooled to room temperature. The amount of sugar released was estimated using a standard curve of d-xylose (Sigma-Aldrich, St. Louis, MO, USA). One unit (U) of xylanase activity was defined as 1 μmol of reducing sugars released from substrate per minute per mL of enzyme under the assay condition. The enzyme activity was calculated by this standard procedure unless otherwise noted. All reactions were performed in at least triplicate.

### 3.5. Biochemical Characterization of XynRA2 and XynRA2ΔCBM

The optimum pH of XynRA2 was determined in a range of 2–11 at 50 °C. The buffers used were 0.1 M of glycine HCl (pH 2–3), sodium acetate (pH 4–6), Tris-HCl (pH 7–9), and glycine-NaOH (pH 10–11) containing 1% (*w*/*v*) purified beechwood xylan (Megazyme, Bray, County Wicklow, Ireland). The optimum temperature of the enzyme was determined over a range of temperature from 20 to 90 °C in Tris-HCl buffer (pH 8.5). The optimum pH of XynRA2ΔCBM was determined at 70 °C and the optimum temperature was determined in acetate buffer (pH 6.0). Thermostability of XynRA2 and XynRA2ΔCBM were determined by measuring the residual activity of the enzyme after pre-incubation in 0.1 M Tris-HCl buffer (pH 8.5) and 0.1 M acetate buffer (pH 6.0), respectively, at 70 °C without substrate for 2 h. The initial activity of enzymes without pre-incubation was set as 100%.

The effect of NaCl on the activity of XynRA2 and XynRA2ΔCBM was determined at 70 °C in 0.1 M Tris-HCl buffer (pH 8.5) and 0.1 M sodium acetate buffer (pH 6.0), respectively, in the presence of up to 5.0 M NaCl. 

To determine the specific activities of purified XynRA2 and XynRA2ΔCBM, the enzyme activities were determined using a xylanase assay as described above, and the protein concentration was determined by Pierce™ BCA Protein Assay kit (Thermo Scientific, Waltham, MA, USA) using BSA as a standard. To determine the turnover rate (*k*_cat_) of XynRA2 and XynRA2ΔCBM, the respective enzymatic reaction was carried out at 70 °C in 0.1 M Tris-HCl buffer (pH 8.5) and 0.1 M sodium acetate buffer (pH 6.0) containing 0.1–1.5% (*w*/*v*) of purified beechwood xylan. The *k*_cat_ of the enzymes were determined based on non-linear regression using PRISM 7 software (GraphPad Software Inc., San Diego, CA, USA).

### 3.6. Analysis of Substrate Specificity and Hydrolysis Products

The substrate specificity of XynRA2 and XynRA2ΔCBM was determined in 0.1 M Tris-HCl (pH 8.5) and 0.1 M acetate buffer (pH 6.0) containing 1% (*w*/*v*) of beechwood xylan, arabinan (Megazyme, Bray, County Wicklow, Ireland), oat-spelt xylan, CMC, Avicel™, D-cellobiose (Sigma, St. Louis, MO, USA), pullulan (TCI chemicals, Tokyo, Japan), starch (QReC, Auckland, New Zealand) or 5 mM of X_2_–X_6_ xylo-oligosaccharides (Bz Oligo Biotech, Qingdao, China). The reaction mixture contained 50 μL of purified enzymes and 500 μL of the substrate solutions. The substrate hydrolysis was detected as described below.

To analyze the hydrolysis products of XynRA2 and XynRA2ΔCBM, the reaction mixtures with 3 U of purified enzymes and 1% (*w*/*v*) beechwood xylan and oat-spelt xylan were incubated at 70 °C in Tris-HCl buffer (pH 8.5) for 24 h. The hydrolysis products were eluted using Rezex™ RSO-oligosaccharides Ag^+^ (4%) column (Phenomenex, Torrance, CA, USA) at a flow rate of 1.0 mL/min at 80 °C for 80 min and detected using 1260 Infinity ELSD (Agilent Technologies, Santa Clara, CA, USA). Xylo- oligosaccharides (X_2_–X_6_) and xylose were used as its product standards.

## 4. Conclusions

*Roseithermus* is a newly proposed genus in family *Rhodothermaceae* affiliated to order *Rhodothermales*. Currently, the whole taxonomic order is comprised of only 14 type strains. So far, xylanase from *Rhodothermus marinus* is the only enzyme that was well characterized. This study described for the first time the biochemical properties of a xylanase from *Roseithermus*. The native XynRA2 was active at alkaline pH and elevated temperature (pH 8.5 and 70 °C) while retaining excellent activity even at 5.0 M NaCl. Such properties make XynRA2 a potential candidate for applications involving an alkaline environment, elevated temperature, and high salinity. In a separate part of the study, the CBM4_9 domain was removed. The data elucidated that CBM truncation affected enzyme specific activity, turnover rate, pH optimum, and NaCl tolerance, with an additional marginal effect on thermostability. 

## Figures and Tables

**Table 1:** The reported halo-tolerant xylanases and their activity in high concentration of NaCl. Enzymatic reactions carried out at 0 M of NaCl was treated as 100%.

Xylanases	Activity in NaCl (%)			Strains	Reference
	3.0 M	4.0 M	5.0 M		
XynRA2	95	94	71	*R. sacchariphilus RA* strain RA	This study
M11	60	50	47	*Streptomyces viridochromogenes*	[12]
Xyn512	60	47	32	*Flammeovirga pacifica* WPAGA1	[23]
XynSL3	60	40	ND	*Alkalibacterium* sp. SL3	[24]
XynA	78	53	ND	*Glaciecola mesophila* KMM 241	[50]
XynA	180	140	100	*Zunongwangia profunda*	[49]
XynSL4	59	41	ND	*Planococcus* sp. SL4	[51]
XynRBM26	96	93	87	*Massilia* sp. RBM26	[52]
XynAHJ3	40	26	ND	*Lechevalieria* sp. HJ3	[53]
Xylanase	90	87	84	*Gracillibacillus* sp. TSCPVG	[54]

ND: not determined.

## Abbreviations

ANI	average nucleotide identity
BCA	bicinchoninic acid
BSA	bovine serum albumin
BLAST	Basic Local Alignment Search Tool
CAZy	carbohydrate active enzymes database
CBM	carbohydrate-binding module
CMC	carboxymethylcellulose
CTD	C-terminal domain
DNS	dinitrosalicylic acid
ELSD	evaporative light scattering detector
GH	glycoside hydrolase
HPLC	high-performance liquid chromatography
IPTG	isopropyl-β-d-1-thiogalactopyranoside
MEGA 7	Molecular Evolutionary Genetics Analysis version 7
MWCO	molecular weight cut-off
NCBI	National Center for Biotechnology Information
Ni-NTA	nickel-nitrilotriacetic acid
PorSS	Por secretion system
SDS-PAGE	sodium dodecyl sulfate-polyacrylamide gel electrophoresis
TIM	triosephosphate isomerase
T9SS	type IX secretion system
XOs	xylo-oligosaccharides
X_1_	xylose
X_2_	xylobiose
X_3_	xylotriose
X_4_	xylotetraose
X_5_	xylopentaose
X_6_	xylohexaose

## References

[1] Collins T., Gerday C., Feller G. (2005). Xylanases, xylanase families and extremophilic xylanases. FEMS Microbiol. Rev..

[2] Moreira L.R.S. (2016). Insights into the mechanism of enzymatic hydrolysis of xylan. Appl. Microbiol. Biotechnol..

[3] Terrapon N., Lombard V., Drula E., Coutinho P.M., Henrissat B., Aoki-Kinoshita K.F. (2017). The CAZy Database/the Carbohydrate-Active Enzyme (CAZy) Database: Principles and Usage Guidelines. A Practical Guide to Using Glycomics Databases.

[4] Nguyen S.T., Freund H.L., Kasanjian J., Berlemont R. (2018). Function, distribution, and annotation of characterized cellulases, xylanases, and chitinases from CAZy. Appl. Microbiol. Biotechnol..

[5] Chakdar H., Kumar M., Pandiyan K., Singh A., Nanjappan K., Kashyap P.L., Srivastava A.K. (2016). Bacterial xylanases: Biology to biotechnology. 3 Biotech.

[6] Juturu V., Wu J.C. (2012). Microbial xylanases: Engineering, production and industrial applications. Biotechnol. Adv..

[7] Kumar V., Marin-Navarro J., Shukla P. (2016). Thermostable microbial xylanases for pulp and paper industries: Trends, applications and further perspectives. World J. Microb. Biotechnol..

[8] Luo H., Wang Y., Li J., Yang J., Yang Y., Huang H., Fan Y., Yao B. (2009). Cloning, expression and characterization of a novel acidic xylanase, XYL11B, from the acidophilic fungus *Bispora* sp. MEY-1. Enzyme Microb. Technol..

[9] Kumar B.K., Balakrishnan H., Rele M. (2004). Compatibility of alkaline xylanases from an alkaliphilic *Bacillus* NCL (87-6-10) with commercial detergents and proteases. J. Ind. Microbiol. Biot..

[10] Dornez E., Verjans P., Arnaut F., Delcour J.A., Courtin C.M. (2011). Use of psychrophilic xylanases provides insight into the xylanase functionality in bread making. J. Agr. Food Chem..

[11] Nagar S., Mittal A., Gupta V.K. (2012). Enzymatic clarification of fruit juices (apple, pineapple, and tomato) using purified *Bacillus pumilus* SV-85S xylanase. Biotechnol. Bioproc. Eng..

[12] Liu Z., Zhao X., Bai F. (2013). Production of xylanase by an alkaline-tolerant marine-derived *Streptomyces viridochromogenes* strain and improvement by ribosome engineering. Appl. Microbiol. Biotechnol..

[13] Talamantes D., Biabini N., Dang H., Abdoun K., Berlemont R. (2016). Natural diversity of cellulases, xylanases, and chitinases in bacteria. Biotechnol. Biofuels.

[14] Chawachart N., Anbarasan S., Turunen S., Li H., Khanongnuch C., Hummel M., Sixta H., Granström T., Lumyong S., Turunen O. (2014). Thermal behaviour and tolerance to ionic liquid [emim]OAc in GH10 xylanase from *Thermoascus aurantiacus* SL16W. Extremophiles.

[15] Evangelista D.E., Kadowaki M.A.S., Mello B.L., Polikarpov I. (2018). Biochemical and biophysical characterization of novel GH10 xylanase prospected from a sugar cane bagasse compost-derived microbial consortia. Int. J. Biol. Macromol..

[16] Niderhaus C., Garrido M., Insani M., Campos E., Wirth S. (2018). Heterologous production and characterization of a thermostable GH10 family endo-xylanase from *Pycnoporus sanguineus* BAFC 2126. Process Biochem..

[17] Sharma K., Antunes I.L., Rajulapati V., Goyal A. (2018). Molecular characterization of a first endo-acting β-1,4-xylanase of family 10 glycoside hydrolase (PsGH10A) from *Pseudopedobacter saltans* comb. nov. Process Biochem..

[18] Guillén D., Sánchez S., Rodríguez-Sanoja R. (2010). Carbohydrate-binding domains: Multiplicity of biological roles. Appl. Microbiol. Biotechnol..

[19] Varnai A., Mäkelä M.R., Djajadi D.T., Rahikainen J., Hatakka A., Viikari L. (2014). Carbohydrate-binding modules of fungal cellulases: Occurrence in nature, function, and relevance in industrial biomass conversion. Advances in Applied Microbiology.

[20] Goh K.M., Chan K.-G., Lim S.W., Liew K.J., Chan C.S., Shamsir M.S., Ee R., Adrian T.-G.-S. (2016). Genome analysis of a new *Rhodothermaceae* strain isolated from a hot spring. Front. Microbiol..

[21] Liew K.J., Teo S.C., Shamsir M.S., Sani R.K., Chong C.S., Chan K.-G., Goh K.M. (2018). Complete genome sequence of *Rhodothermaceae* bacterium RA with cellulolytic and xylanolytic activities. 3 Biotech.

[22] Park M.-J., Oh J.H., Yang S.-H., Kwon K.K. (2019). *Roseithermus sacchariphilus* gen. nov., sp. nov. and proposal of *Salisaetaceae* fam. nov., representing new family in the order *Rhodothermales*. Int. J. Syst. Evol. Microbiol..

[23] Cai Z.-W., Ge H.-H., Yi Z.-W., Zeng R.-Y., Zhang G.-Y. (2018). Characterization of a novel psychrophilic and halophilic β-1,3-xylanase from deep-sea bacterium, *Flammeovirga pacifica* strain WPAGA1. Int. J. Biol. Macromol..

[24] Wang G., Wu J., Yan R., Lin J., Ye X. (2017). A novel multi-domain high molecular, salt-stable alkaline xylanase from *Alkalibacterium* sp. SL3. Front. Microbiol..

[25] Yadav P., Maharjan J., Korpole S., Prasad G.S., Sahni G., Bhattarai T., Sreerama L. (2018). Production, purification, and characterization of thermostable alkaline xylanase from *Anoxybacillus kamchatkensis* NASTPD13. Front. Bioeng. Biotechnol..

[26] Chan C.S., Chan K.-G., Ee R., Hong K.-W., Urbieta M.S., Donati E.R., Shamsir M.S., Goh K.M. (2017). Effects of physiochemical factors on prokaryotic biodiversity in Malaysian circumneutral hot springs. Front. Microbiol..

[27] Karlsson E.N., Bartonek-Roxå E., Holst O. (1997). Cloning and sequence of a thermostable multidomain xylanase from the bacterium *Rhodothermus marinus*. BBA - Gene Struct. Expr..

[28] Rakitin A.L., Ermakova A.Y., Ravin N.V. (2015). Novel endoxylanases of the moderately thermophilic polysaccharide-degrading bacterium *Melioribacter roseus*. J. Microbiol. Biotechnol..

[29] Beliën T., Van Campenhout S., Van Acker M., Volckaert G. (2005). Cloning and characterization of two endoxylanases from the cereal phytopathogen *Fusarium graminearum* and their inhibition profile against endoxylanase inhibitors from wheat. Biochem. Biophys. Res. Commun..

[30] La Grange D.C., Pretorius I.S., Van Zyl W.H. (1996). Expression of a *Trichoderma reesei* beta-xylanase gene (XYN2) in *Saccharomyces cerevisiae*. Appl. Environ. Microbiol..

[31] Khasin A., Alchanati I., Shoham Y. (1993). Purification and characterization of a thermostable xylanase from *Bacillus stearothermophilus* T-6. Appl. Environ. Microbiol..

[32] Lee Y.-E., Lowe S., Henrissat B., Zeikus J.G. (1993). Characterization of the active site and thermostability regions of endoxylanase from *Thermoanaerobacterium saccharolyticum* B6A-RI. J. Bacteriol..

[33] Lasica A.M., Ksiazek M., Madej M., Potempa J. (2017). The type IX secretion system (T9SS): Highlights and recent insights into its structure and function. Front. Cell. Infect. Microbiol..

[34] Sato K., Yukitake H., Narita Y., Shoji M., Naito M., Nakayama K. (2013). Identification of *Porphyromonas gingivalis* proteins secreted by the Por secretion system. FEMS Microbiol. Lett..

[35] Karlsson E.N., Hachem M.A., Ramchuran S., Costa H., Holst O., Svenningsen Å.F., Hreggvidsson G.O. (2004). The modular xylanase Xyn10A from *Rhodothermus marinus* is cell-attached, and its C-terminal domain has several putative homologues among cell-attached proteins within the phylum *Bacteroidetes*. FEMS Microbiol. Lett..

[36] Kharade S.S., McBride M.J. (2014). *Flavobacterium johnsoniae* chitinase ChiA is required for chitin utilization and is secreted by the type IX secretion system. J. Bacteriol..

[37] Veith P.D., Nor Muhammad N.A., Dashper S.G., Likić V.A., Gorasia D.G., Chen D., Byrne S.J., Catmull D.V., Reynolds E.C. (2013). Protein substrates of a novel secretion system are numerous in the *Bacteroidetes* phylum and have in common a cleavable C-terminal secretion signal, extensive post-translational modification, and cell-surface attachment. J. Proteome Res..

[38] Hachem M.A., Karlsson E.N., Bartonek-Roxå E., Raghothama S., Simpson P.J., Gilbert H.J., Williamson M.P., Holst O. (2000). Carbohydrate-binding modules from a thermostable *Rhodothermus marinus* xylanase: Cloning, expression and binding studies. Biochem. J..

[39] Liu Y., Huang L., Li W., Guo W., Zheng H., Wang J., Lu F. (2015). Studies on properties of the xylan-binding domain and linker sequence of xylanase XynG1-1 from *Paenibacillus campinasensis* G1-1. J. Ind. Microbiol. Biot..

[40] Li N., Shi P., Yang P., Wang Y., Luo H., Bai Y., Zhou Z., Yao B. (2009). A xylanase with high pH stability from *Streptomyces* sp. S27 and its carbohydrate-binding module with/without linker-region-truncated versions. Appl. Microbiol. Biotechnol..

[41] Zhang M., Chekan J.R., Dodd D., Hong P.-Y., Radlinski L., Revindran V., Nair S.K., Mackie R.I., Cann I. (2014). Xylan utilization in human gut commensal bacteria is orchestrated by unique modular organization of polysaccharide-degrading enzymes. Proc. Natl. Acad. Sci. USA.

[42] Karlsson E.N., Bartonek-Roxå E., Holst O. (1998). Evidence for substrate binding of a recombinant thermostable xylanase originating from *Rhodothermus marinus*. FEMS Microbiol. Lett..

[43] Li Q., Sun B., Li X., Xiong K., Xu Y., Yang R., Hou J., Teng C. (2018). Improvement of the catalytic characteristics of a salt-tolerant GH10 xylanase from *Streptomyce rochei* L10904. Int. J. Biol. Macromol..

[44] Lim H.K., Lee K.I., Hwang I.T. (2016). Identification of a novel cellulose-binding domain within the endo-β-1,4-xylanase KRICT PX-3 from *Paenibacillus terrae* HPL-003. Enzyme Microb. Technol..

[45] Karlsson E.N., Dahlberg L., Torto N., Gorton L., Holst O. (1998). Enzymatic specificity and hydrolysis pattern of the catalytic domain of the xylanase Xyn1 from *Rhodothermus marinus*. J. Biotechnol..

[46] Hachem M.A., Olsson F., Nordberg Karlsson E. (2003). Probing the stability of the modular family 10 xylanase from *Rhodothermus marinus*. Extremophiles.

[47] Graziano G., Merlino A. (2014). Molecular bases of protein halotolerance. BBA - Proteins Proteom..

[48] Fukuchi S., Yoshimune K., Wakayama M., Moriguchi M., Nishikawa K. (2003). Unique amino acid composition of proteins in halophilic bacteria. J. Mol. Biol..

[49] Liu X., Huang Z., Zhang X., Shao Z., Liu Z. (2014). Cloning, expression and characterization of a novel cold-active and halophilic xylanase from *Zunongwangia profunda*. Extremophiles.

[50] Guo B., Chen X.-L., Sun C.-Y., Zhou B.-C., Zhang Y.-Z. (2009). Gene cloning, expression and characterization of a new cold-active and salt-tolerant endo-β-1,4-xylanase from marine *Glaciecola mesophila* KMM 241. Appl. Microbiol. Biotechnol..

[51] Huang X., Lin J., Ye X., Wang G. (2015). Molecular characterization of a thermophilic and salt-and alkaline-tolerant xylanase from *Planococcus* sp. SL4, a strain isolated from the sediment of a soda lake. J. Microbiol. Biotechnol..

[52] Xu B., Dai L., Li J., Deng M., Miao H., Zhou J., Mu Y., Wu Q., Tang X., Yang Y. (2015). Molecular and biochemical characterization of a novel xylanase from *Massilia* sp. RBM26 isolated from the feces of *Rhinopithecus bieti*. J. Microbiol. Biotechnol..

[53] Zhou J., Gao Y., Dong Y., Tang X., Li J., Xu B., Mu Y., Wu Q., Huang Z. (2012). A novel xylanase with tolerance to ethanol, salt, protease, SDS, heat, and alkali from actinomycete *Lechevalieria* sp. HJ3. J. Ind. Microbiol. Biot..

[54] Poosarla V.G., Chandra T. (2014). Purification and characterization of novel halo-acid-alkali-thermo-stable xylanase from *Gracilibacillus* sp. TSCPVG. Appl. Biochem. Biotech..

[55] Verjans P., Dornez E., Segers M., Van Campenhout S., Bernaerts K., Beliën T., Delcour J.A., Courtin C.M. (2010). Truncated derivatives of a multidomain thermophilic glycosyl hydrolase family 10 xylanase from *Thermotoga maritima* reveal structure related activity profiles and substrate hydrolysis patterns. J. Biotechnol..

[56] Sunna A., Gibbs M.D., Bergquist P.L. (2000). A novel thermostable multidomain 1,4-β-xylanase from ‘*Caldibacillus cellulovorans*’ and effect of its xylan-binding domain on enzyme activity. Microbiol..

[57] Ali M.K., Hayashi H., Karita S., Goto M., Kimura T., Sakka K., Ohmiya K. (2001). Importance of the carbohydrate-binding module of *Clostridium stercorarium* Xyn10B to xylan hydrolysis. Biosci. Biotechnol. Biochem..

[58] Bai W., Xue Y., Zhou C., Ma Y. (2015). Cloning, expression, and characterization of a novel alkali-tolerant xylanase from alkaliphilic *Bacillus* sp. SN5. Biotechnol. Appl. Biochem..

[59] Kumar S., Stecher G., Tamura K. (2016). MEGA7: Molecular evolutionary genetics analysis version 7.0 for bigger datasets. Mol. Biol. Evol..

[60] Gullfot F., Tan T.-C., von Schantz L., Karlsson E.N., Ohlin M., Brumer H., Divne C. (2010). The crystal structure of XG-34, an evolved xyloglucan-specific carbohydrate-binding module. Proteins.

[61] Santos C.R., Hoffmam Z.B., de Matos Martins V.P., Zanphorlin L.M., de Paula Assis L.H., Honorato R.V., de Oliveira P.S.L., Ruller R., Murakami M.T. (2014). Molecular mechanisms associated with xylan degradation by *Xanthomonas* plant pathogens. J. Biol. Chem..

